# Ultrasonographic Gallbladder Abnormality of Primary Epstein–Barr Virus Infection in Children and Its Influence on Clinical Outcome

**DOI:** 10.1097/MD.0000000000001120

**Published:** 2015-07-13

**Authors:** Dae Yong Yi, Ji Young Kim, Hye Ran Yang

**Affiliations:** From Department of Pediatrics, Seoul National University Bundang Hospital, Seongnam, Gyeonggi-do, Republic of Korea (DYY, HRY); Department of Pediatrics, Chung-Ang University Hospital, Seoul, Republic of Korea (DYY); Department of Radiology, Seoul National University Bundang Hospital, Seongnam, Gyeonggi-do, Republic of Korea (JYK); and Seoul National University College of Medicine, Seoul, Republic of Korea (HRY).

## Abstract

The incidence of pediatric acute inflammatory gallbladder (GB) disease without gallstone such as acute acalculous cholecystitis has increased with the development of improved diagnostic modalities. Although Epstein–Barr virus (EBV) infection is common in general population, only few cases of GB diseases caused by EBV infection have been reported. This study analyzed ultrasonographic characteristics of primary EBV infection in children and evaluated the influence of EBV-associated GB disease on clinical course and outcome of EBV infection.

Between March 2004 and January 2013, 94 of 287 pediatric patients with EBV infection underwent abdominal ultrasonography (USG); clinical features, laboratory data, and USG findings were collected and analyzed retrospectively.

Of 94 children, ultrasonographic thick GB wall was observed in 24 (25.3%). Platelet counts were lower in the thickened GB wall group than in the normal GB wall thickness group (*P* = 0.004). Direct bilirubin, alanine aminotransferase, and γ-glutamyl transferase levels were higher in the thickened GB wall group (*P* = 0.000, *P* = 0.041, and *P* = 0.001, respectively). The duration of hospitalization was longer in patients with thickened GB wall (*P* = 0.043).

Radiologic findings of acute acalculous inflammatory GB disease such as thickened GB wall caused by primary EBV infection are more common than previously reported. Consideration of EBV infection in the differential diagnosis of children suspected with acute acalculous GB diseases may avoid unnecessary surgical intervention.

## INTRODUCTION

Because the incidence of acute inflammatory gallbladder (GB) disease without gallstone such as acute acalculous cholecystitis (AAC) in pediatric population is low, acute acalculous GB diseases are not usually included in the differential diagnosis for children with complaints of gastrointestinal (GI) symptoms such as abdominal pain and vomiting.^1^ However, with the development of diagnostic modalities such as abdominal ultrasonography (USG) and with increased attention to this condition, the prevalence of acute inflammatory GB disease without gallstone is rising in children.^2–4^ The prognosis of acute acalculous GB disease is generally good, but when the diagnosis is delayed or incorrect, complications such as perforation and gangrene can occur, necessitating surgical intervention. Therefore, prompt and accurate diagnosis and treatment is important.^3,5–7^

The cause of acute acalculous GB disease in pediatric patients is not yet clear. The disease may be secondary to systemic disorders such as Kawasaki disease, burns, vascular disease, or metabolic disease.^3,7,8^ It can also be caused by systemic infections, including streptococci, Gram-negative organisms, hepatitis A virus, or Epstein–Barr virus (EBV) infection.^3,6,9,10^

Children with EBV infection usually have nonspecific symptoms or are asymptomatic. However, clinical manifestation of infectious mononucleosis including hepatosplenomegaly, and hepatobiliary dysfunction may occur.^11,12^ Occasionally, variable diseases such as lymphoma and fatal complications may be associated to EBV infection.^11^ EBV infection has been suggested to be important in the etiology of acute inflammatory GB disease, but there have been only several case reports so far, and no studies on the clinical course or prognosis of childhood primary EBV infection complicated by acute inflammatory GB disease.^9–11,13^

Therefore, this study aimed to analyze the ultrasonographic characteristics of primary EBV infection in children, as well as to evaluate the influence of EBV-associated GB a disease on clinical course of primary EBV infection.

## SUBJECTS AND METHODS

### Patients and Data Extraction

Of all patients presenting to Seoul National University Bundang Hospital due to hepatobiliary manifestations such as abdominal pain, jaundice, and liver function test (LFT) abnormalities between March 2004 and February 2014, there were 287 pediatric patients diagnosed with EBV infection who were initially recruited for the study. The diagnosis of EBV infection was based on a positive EBV viral capsid antigen (VCA) IgM antibodies and the presence of clinical manifestations compatible with primary EBV infection. We included patients who were healthy before hospital admission and excluded patients with hepatobiliary dysfunction due to other underlying diseases (ie, other systemic infections, Kawasaki disease, malignancy or trauma, etc.) from the study. We also excluded the patients who did not undergo abdominal USG during the clinical course of primary EBV infection. Of the 287 patients, 94 subjects were finally included, and the abdominal USG, clinical features, and laboratory data retrospectively reviewed. Those 94 patients were classified into 2 groups according to the presence or absence of ultrasonographic abnormalities of the GB.

The subjects were also categorized into the 4 groups according to age classifications developed by the Eunice Kennedy Shriver National Institute of Child Health and Human Development: infant (0–2 years), early childhood (2–5 years), middle childhood (6–11 years), and early adolescence (12–18 years).^14^

This study was conducted with the approval of the Institutional Review Board of the Seoul National University Bundang Hospital (IRB No.: B-1412-280-103).

### Laboratory Tests and Diagnosis of EBV Infection

For all subjects, LFTs including total and direct bilirubin, serum aminotransferases, and γ glutamyl transferase (γGT) were obtained, as were routine laboratory tests including white blood cell count (WBC), hemoglobin, hematocrit, platelet count, and highly sensitive C-reactive protein (hsCRP). The duration of hospital stay due to abdominal pain, vomiting, jaundice, and pyrexia was also documented.

Patients were defined as having EBV infection if they had a positive EBV VCA IgM in the acute phase of the disease. EBV VCA IgM antibody was detected using an enzyme-linked immunosorbent assay (ELISA) in vitro diagnostic device for the quantitative measurement of IgM antibodies against the VCA antigens p23 and p18 of EBV. Serum samples of patients were used for the diagnosis, and tested with the anti-EBV VCA ELISA kit (Thunderbolt^®^, Korea).

### Ultrasonographic Diagnosis of Acalculous Inflammatory GB Disease

Abdominal USG was performed by expert pediatric radiologists during the acute stage of EBV infection. Initial USG findings and interpretations were retrospectively reviewed by another expert pediatrics radiologist as well as an expert pediatric GI-hepatologist. The ultrasonographic diagnosis of acute inflammatory GB disease was made based on 4 diagnostic USG criteria of AAC: GB distention, GB wall thickness greater than 3.5 mm, nonshadowing echogenic sludge, and pericholecystic fluid collections.^15^

### Statistical Analysis

Statistical analysis was performed using SPSS 18.0 statistical software (SPSS, Inc., Chicago, IL). Student t test, Pearson's χ^2^ test, and Mann–Whitney *U* test were used to evaluate the differences between the groups. The level of statistical significance was set at *P* < 0.05.

## RESULTS

The clinical manifestations and ultrasonographic findings of our pediatric patients with EBV infection are listed in Table 1. Of 94 children with EBV infection, the mean age was 7.1 ± 4.3 years old, 47 were boys and 47 were girls. Ultrasonographic thick GB wall than 3.5 mm was found in 24 (25.3%), comprising the thickened GB wall group. Pericholecystic fluid was also identified in 2 of these patients. GB distension and GB sludge were not identified in any patients. Five patients (5.3%) were hospitalized due to hypotension or septic shock with infection in intensive care unit (ICU) for an average of 13 (range: 1–22) days.

**TABLE 1:**
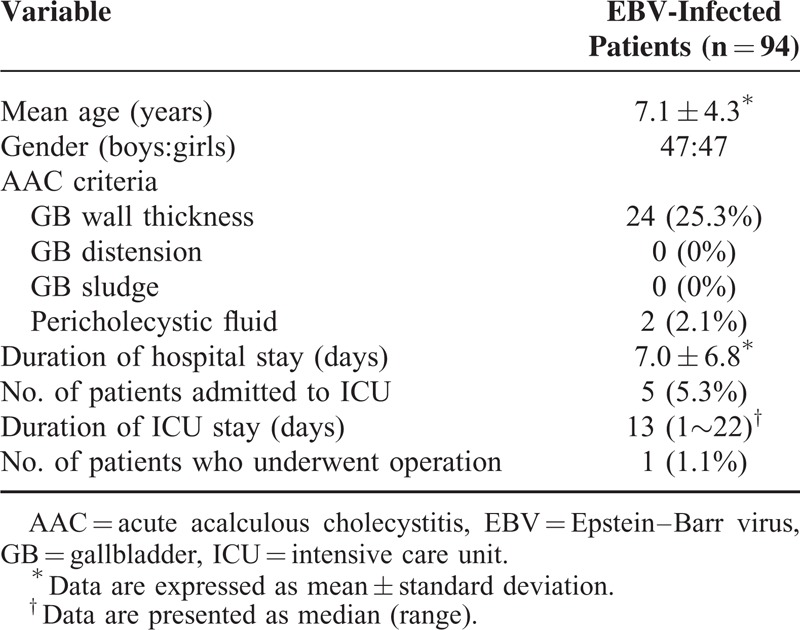
Clinical Manifestations and Sonographic Findings in Pediatric Patients With Epstein–Barr Virus Infection

Of the 94 subjects, only 1 patient—a 16-year-old female adolescent—underwent an operation (Table 1). This patient presented to an emergency room with acute right upper quadrant abdominal pain and pyrexia, and computed tomography of the abdomen showed findings partially compatible with AAC presented prominent thickened GB wall. The patient underwent emergency surgery. Abnormal LFT results persisted postoperatively, and subsequent evaluation by a pediatric hepatologist revealed a positive EBV VCA IgM, for which further supportive management was provided.

**FIGURE 1:**
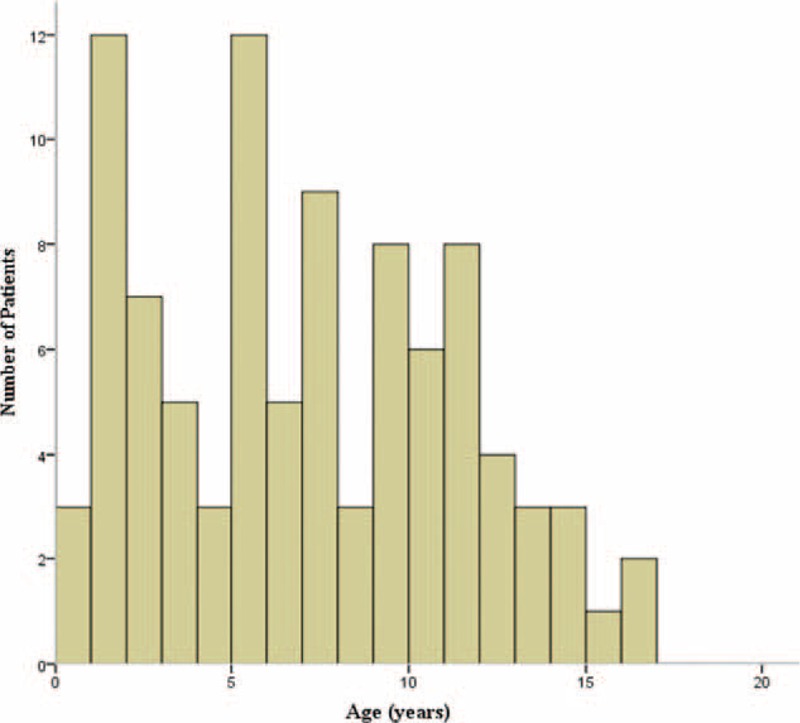
Age distribution in pediatric patients with Epstein–Barr virus (EBV) infection. The graph indicates that EBV infection was relatively prevalent in early and middle childhood compared to other age groups.

Figure 1 shows that EBV infection was most prevalent from early childhood through middle childhood in our subjects. In comparing the clinical manifestations and ultrasonographic findings according to age group, no significant differences in sex, duration of hospital stay, and duration of ICU stay (*P* = 0.655, *P* = 0.673, and *P* = 0.952, respectively) were found among the 4 age groups. Thickened GB wall on USG was more frequently noted in childhood and adolescent groups than the infancy group, but this was not statistically significant (*P* = 0.064).

Clinical features of pediatric patients with EBV infection were compared to the presence of ultrasonographic GB disease (Table 2). There were no statistically significant differences between the thickened GB wall group and normal GB wall group with regard to age, sex, or number of patients (*P* = 0.813, *P* = 0.478, and *P* = 0.599, respectively) who required admission to the ICU. However, the thickened GB wall group had significantly longer hospitalizations than the normal GB wall group (*P* = 0.043) (Table 2). When laboratory findings of pediatric patients with EBV infection were compared according to ultrasonographic GB disease, platelet count was significantly lower in the thickened GB wall group than in the normal GB wall group (*P* = 0.004), whereas the levels of serum direct bilirubin, alanine aminotransferase, and γGT were significantly higher (*P* = 0.000, *P* = 0.041, and *P* = 0.001, respectively) in the thickened GB wall group (Table 3). There were no significant differences in the levels of hemoglobin, WBC count, and hsCRP (*P* = 0.829, *P* = 0.288, *P* = 0.881, respectively) (Table 3).

**TABLE 2:**
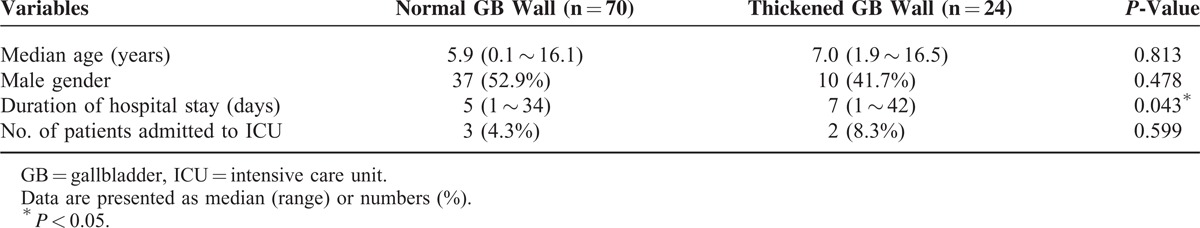
Comparison of Clinical Features in Pediatric Patients With Epstein–Barr Virus Infection According to the Sonographic Gallbladder Wall Thickness

**TABLE 3:**
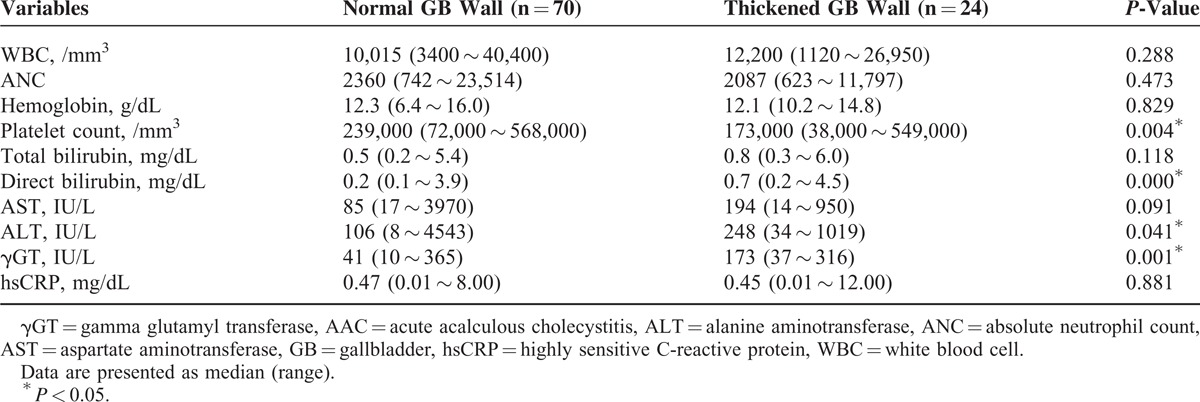
Comparison of Laboratory Findings in Pediatric Patients With Epstein–Barr Virus Infection According to the Sonographic Gallbladder Wall Thickness

Additional abdominal ultrasonographic findings in pediatric patients with EBV infection were compared with ultrasonographic GB abnormalities (Table 4). GB abnormality on USG was more frequently observed in the thickened GB wall group than in the normal GB wall group. Both hepatomegaly and splenomegaly were more frequently noted in the thickened GB wall group than in the normal GB wall group (*P* = 0.003 and *P* = 0.001). The presence of increased periportal echogenicity and periportal lymph nodes were also significantly more frequent in the thickened GB wall group than in the normal GB wall group (*P* = 0.000 and *P* = 0.039).

**TABLE 4:**
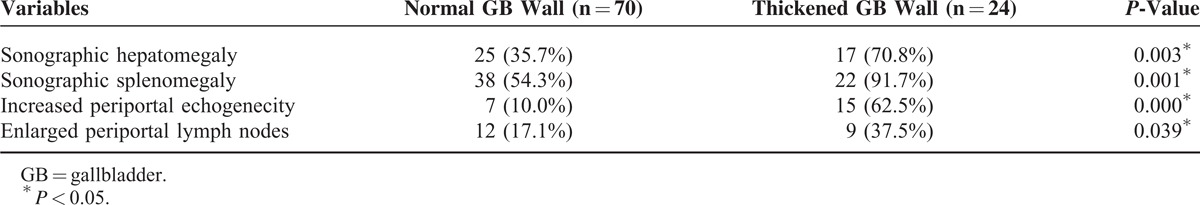
Comparison of Additional Abdominal Sonographic Findings in Pediatric Patients With Epstein–Barr Virus Infection According to the Sonographic Gallbladder Wall Thickness

## DISCUSSION

In adults, acute cholecystitis is often due to gallstones and requires surgical treatment such as cholecystectomy. In contrast, acute inflammatory GB disease without gallstone such as AAC caused by predisposing diseases process accounts for a large portion (30–50%) of all pediatric acute cholecystitis cases.^16,17^

Some studies involving adult patients have proposed that GB disease without gallstone may need early operation^7,18,19^; whether early surgical intervention is necessary for children still remains controversial. Recent reports suggest that acute inflammatory GB disease in children and adolescents is caused by a variety of conditions other than gallstones, and a nonsurgical approach is preferred.^7–9,15^ Huang and Yang^3^ reported that they approach all AAC patients with nonsurgical management, despite a higher mortality rate due to septic shock and hypofibrinogenemia. It may thus be important to identify the exact cause of acute cholecystitis in children in order to predict its clinical course and prognosis.

Although EBV is considered one of the possible causes of acute inflammatory GB disease, only a few case reports exist to date, and there is no research on its clinical course or USG findings.^9–11,13^ In the present study over a 10-year period, 24 out of 94 pediatric patients with a primary EBV infection were found to have a significantly thickened GB wall during the acute phase of the illness. This suggests that sonographic acute GB abnormality associated with EBV infection may be far more common in children than previously reported.

The age distribution of children and adolescents with EBV infection in our study shows that EBV infection can occur at relatively young age, especially in early and middle childhood rather than adolescence, compatible with a previous report that EBV infection occurs more commonly during early childhood in industrialized countries.^20^ According to previous reports, primary EBV infection with LFT abnormalities occurs mostly before the age of 25 years, and about one-third of cases occur during adolescence.^9,10,20^

Acute inflammatory GB disease without gallstone in pediatric patients is more frequent in boys, unlike calculous cholecystitis in adults, which has a slight predominance in women.^9,21,22^ On the other hand, EBV-associated acute GB disease is known to occur slightly more frequently in women.^9^ Sex distribution in our study revealed a similar female predominance, but this difference was not significant. Our findings cannot be generalized because of the small sample size, and supplemental data from other regions and institutions are needed.

Previous reports on the prognosis of acute inflammatory GB disease without gallstone reveal a variety of results, and data on the outcome of EBV-associated GB disease is lacking. Some case reports suggest that EBV-associated GB disease may have a relatively favorable prognosis.^9–11,13^ In the present study, the overall hospital stay of the EBV-infected patients with thickened GB wall on USG was significantly longer. However, the number of patients transferred to the ICU was not significantly different between the thickened GB wall and the normal GB wall groups, and there were no patients with critical complications such as GB perforation or death in our study. Only 1 patient with cholecystitis underwent emergent surgery. This patient was later diagnosed with AAC and cholestatic hepatitis caused by EBV infection. Although early surgical intervention was of questionable benefit and somewhat controversial for this patient, the rest of the study subjects with AAC improved with supportive medical treatment. Clinicians should be aware of EBV-associated AAC in pediatric patients to avoid unnecessary surgery and to consider appropriate conservative management.

In our previous study, we found that GB distension was the most common sonographic GB abnormality in patients with Kawasaki disease.^8^ In contrast, GB distension was not found in any patients with EBV-associated GB disease, whereas thickened GB wall was prominently observed. Although the pathogenesis of this finding is not certain, direct invasion of the GB wall might be one of possible mechanisms for GB abnormality caused by EBV infection.^9,23^ Mourani et al described that viral antigen was detected immunohistochemically in the walls of removed GB in hepatitis A virus-associated GB abnormality.^9,23,24^ Previous studies also reported that the homozygous or heterozygous UGT1A1^∗^28 mutation in Gilbert syndrome was related to impaired liver function and increased risk of cholestasis in patients with EBV infection.^25,26^ This mechanism was not proven in EBV- or other virus-associated GB abnormality, but it is likely due to a similar pathogenesis.

Abnormal laboratory markers including a low platelet count and increased direct bilirubin, alanine aminotransferase, and γGT occurred more often in the EBV-infected patients with thickened GB wall. Previous studies described that ultrasonographic thickened GB wall occurred during acute EBV hepatitis or the course of infectious mononucleosis syndromes, and they proposed GB wall thickening as a sign of severity of the illness.^27,28^ As these findings reflect a relatively poor clinical course, more attention to diagnosis and treatment may be necessary. Furthermore, measuring platelet count, direct bilirubin, alanine aminotransferase, and γGT levels in pediatric EBV patients may aid in the early diagnosis and proper treatment of EBV-associated GB disease.

USG is a useful tool to evaluate suspected GB diseases because it is highly sensitive, provides quick results, and is easy to repeat.^1,6,7^ Nonprimary acute inflammatory GB disease caused by predisposing diseases may be accompanied by additional secondary findings on USG, and our study revealed that hepatomegaly, splenomegaly, increased periportal echogenicity, and periportal lymph nodes were significantly more frequent in the thickened GB wall group. These findings may be secondary to systemic inflammation affecting the GB and the liver, suggesting that a thorough evaluation of the GB is indicated to confirm the presence of acute inflammatory GB disease in EBV-infected children.

Our study has several limitations. Additional data and daily measurement of liver enzymes would have been useful to compare the prognosis between the thickened GB wall group and normal GB wall group. By necessity, our patients’ LFTs were measured in the outpatient clinic every few weeks or months, making it difficult to pinpoint when LFTs became normal. In addition, the study subjects excluded patients without EBV serology or an abdominal USG despite LFT abnormality at diagnosis. Because these patients improved quickly and their clinical course was uncomplicated, additional viral studies and abdominal USG were not necessarily indicated. These cases may not have influenced our research results significantly; however, it would be beneficial for future prospective studies to include performance of an abdominal USG for all patients with suspected EBV infection.

Despite its limitations, our study is significant as it is the first to compare and analyze ultrasonographic characteristics of primary EBV infection in children and to evaluate the influence of EBV-associated GB disease on clinical course of EBV infection. Our results show that EBV-associated GB disease is more common than previously reported, and although there was a wide range of age distribution, most cases occurred in the early and middle childhood age groups. Although the patients with EBV-associated GB disease generally had a good outcome with few complications, their prognosis was worse than that of the normal GB wall patients. Supportive medical care without surgical intervention resulted in resolution of EBV-associated GB disease in most pediatric cases.

## CONCLUSION

The results of the present study suggest that ultrasonographic abnormalities of the GB caused by primary EBV infection in childhood are more frequent than previously reported. When a pediatric patient presents with acute inflammatory GB disease without gallstone, EBV infection must be included in the differential diagnosis to prevent potentially unnecessary surgery. Timely decision-making improves both the therapeutic approach and clinical outcome of EBV-infected children and adolescents with acute inflammatory GB disease.
